# Evaluation of Post‐Endodontic Pain Reduction Using Intracanal Cryotherapy in Symptomatic Apical Periodontitis

**DOI:** 10.1111/aej.12983

**Published:** 2025-07-25

**Authors:** Anam Fayyaz Bashir, Ussamah Waheed Jatala, Muhammad Amber Fareed, Sheryar Sheryar, Saadia Ahmad Chattha, Saima Razaq Khan, Shahzad Ahmad, Shazia Iqbal, Muhammad Sohail Zafar, Shahzad Ali

**Affiliations:** ^1^ Department of Operative Dentistry Lahore Medical and Dental College Lahore Pakistan; ^2^ Department of Prosthodontics Lahore Medical and Dental College Lahore Pakistan; ^3^ Clinical Sciences Department College of Dentistry Ajman University Ajman UAE; ^4^ Center of Medical and Bio‐Allied Health Sciences Research Ajman University Ajman UAE; ^5^ Department of Operative Dentistry Rahbar College of Dentistry Lahore Pakistan; ^6^ Faculty of Medicine and Health Sciences The University of Buckingham Buckingham UK; ^7^ College of Dentistry University of Jordan Amman Jordan; ^8^ Department of Pharmacy and Biotechnology Alma Mater Studiorum – Università di Bologna Bologna Italy

**Keywords:** apical periodontitis, cryotherapy, endodontics, postoperative pain, root canal therapy

## Abstract

This prospective experimental study evaluated the efficacy of intracanal cryotherapy in reducing postoperative pain in patients with symptomatic apical periodontitis. Ninety participants with necrotic pulp and preoperative pain (VAS ≥ 7) were randomised into two groups: Group A received room‐temperature saline irrigation, while Group B received cryotreated saline (2.5°C) as the final irrigant. Pain was assessed preoperatively, at 24 h, and 48 h post‐treatment using the Visual Analogue Scale. Results demonstrated significantly lower pain scores in the cryotherapy group at 24 h (*p* = 0.010) and 48 h (*p* < 0.001), with reduced analgesic use. Cryotherapy effectively mitigates post‐operative pain in symptomatic apical periodontitis, offering a non‐invasive adjunct to standard endodontic therapy.

## 1 Introduction

Patient satisfaction and treatment outcomes are greatly impacted by dental pain, which is the main reason people seek dental care [1]. Postoperative pain (POP) is a serious barrier in endodontic therapy. According to research, the prevalence of POP varies between 3% and 58%, depending on variables such as pulp state, preoperative pain severity, and treatment techniques [2, 3, 4]. Microorganisms enter the root canal, usually through caries, and if not removed timely, enter the periapical tissues through the apical portion of the root canal. When the microorganisms exit the canal and enter the periapical area, this causes an inflammatory reaction leading to apical periodontitis. In addition, periapical inflammation can be brought on by chemicals and endodontic materials, unintentional trauma, or injury sustained after root canal instrumentation [5]. Clinically, symptomatic apical periodontitis typically presents as discomfort, tooth elevation in the socket, and tenderness on percussion [6].

Applying freezing temperatures to alleviate pain and inflammation is known as cryotherapy, and it has become a viable adjunct in endodontic therapy. Cryotherapy reduces tissue metabolism and causes local vasoconstriction, which prevents the production of pro‐inflammatory mediators and reduces pain and swelling, thereby reducing inflammation [7, 8]. This technique has been well established in most medical fields, such as orthopaedics and general surgery, for its effectiveness in controlling POP and enhancing recovery [1, 7, 9, 10].

In 2015, Vera et al. introduced cryotherapy to endodontics and demonstrated that the exterior root surface temperature decreased and remained so for four minutes following cryotherapy [11]. Intracanal cryotherapy, which delivers cold saline as a final irrigant for five minutes, has been linked to decreased POP and analgesic intake, especially in dentition with necrotic pulps and symptomatic apical periodontitis [12].

Intracanal cryotherapy has been shown in studies to substantially reduce POP after root canal therapy. Shah et al. found that patients receiving cold saline irrigation reported significantly less discomfort 24 h after single‐visit endodontics compared to those receiving room‐temperature saline [13]. According to a meta‐analysis and systematic review by Hespanhol et al., patients with symptomatic apical periodontitis who received cold saline irrigation reported less discomfort six and twenty‐four hours after therapy than those who received room‐temperature saline [14]. These results are further supported by Ezzat et al.'s findings, which indicate that patients receiving intracanal cryotherapy as the final irrigant experienced a reduction in pain levels [7]. The physiological mechanism behind this effect involves local cooling of the periapical area, deactivating nociceptive pathways and preventing A‐delta fibres and C‐fibres from conducting pain signals [7, 9]. Furthermore, the benefits of cryotherapy go beyond just reducing pain. According to research, it may diminish inflammatory reactions in periapical tissues, potentially aiding healing by reducing pain and inflammation [2, 15].

Although cryotherapy presents significant potential, its implementation in endodontics faces several challenges [16]. The inconsistency in protocols, such as the selection and quantity of cryoagents utilised and the duration of their application, underscores the necessity for standardised practices. Additional research is essential to confirm its long‐term impact on postoperative recovery and to develop universal guidelines for its incorporation into standard clinical procedures.

The aim of this research is to evaluate whether intracanal cryotherapy can effectively reduce POP in patients diagnosed with symptomatic apical periodontitis. Specifically, the objective includes comparing pain levels between patients undergoing cryotherapy and those treated with room temperature saline. In order to improve patient care and endodontic treatment results, this study aims to offer evidence‐based suggestions for using cryotherapy in clinical practice.

## 2 Material and Methods

This prospective experimental study was conducted from March 2024 to August 2024 in the Operative Dentistry department at Lahore Medical and Dental College, Lahore. The study was approved by the college's Institutional Review Board (IRB) under approval number LMDC/FD/5301/23. All participants gave their informed consent prior to study enrollment.

Patients aged 18–60 years, presenting single canal maxillary anteriors, with a diagnosis of necrotic pulp and symptomatic apical periodontitis, were included. Eligibility criteria required a preoperative pain (preopVAS) score of 7–10 on the Visual Analogue Scale (VAS) for 10 days or less. Diagnosis was confirmed through clinical and radiographic evaluation, including percussion testing and an electric pulp tester. Exclusion criteria included patients with systemic diseases (e.g., diabetes, autoimmune illnesses), severe periodontal disease, incomplete apex formation, necrotic teeth, re‐endodontic cases, pregnancy, or use of antibiotics within 10 days prior to treatment.

The experimental group's mean pain scores were 1.85 (SD = 1.81) and the control groups were 3.65 (SD = 3.03), which were used to determine the sample size [8]. With a significance level of 5% and a power of 80%, the necessary sample size was determined to be 31 participants per group, for a total of 62. The final adjusted sample size was raised to 90 participants, with 45 members per group, to account for a 30% dropout rate.

Using computer‐generated randomisation (www.randomizer.org), patients who satisfied the inclusion requirements were divided into two groups at random: Group A (Control) received 20 mL of room‐temperature saline as the last irrigant, while Group B (Experimental) received 20 mL of cryotreated saline at 2.5°C as the last irrigant. Consecutively ordered opaque, taped envelopes were used for group allocation concealment. Although the doctors could not be blindfolded because of the temperature changes in the irrigation solutions, participants were blinded to the intervention.

The treatment protocol began with a preoperative assessment, during which preoperative pain levels were recorded using the Visual Analog Scale (VAS) extending from 0 to 10 where 0 was no pain and 10 was unbearable pain. Local anaesthesia (2% lidocaine with 1:100000 epinephrine) was administered, and a rubber dam was placed. Conventional access cavities were prepared, and the working length was established using an apex locator and verified radiographically. For instrumentation and irrigation, root canals were prepared using ProTaper hand files with intermittent irrigation of 5.25% sodium hypochlorite using a 27‐gauge side‐vented needle. After shaping, canals were flushed with 17% EDTA for 1 min. The intervention phase involved two groups: Group A received final irrigation with 20 mL of room‐temperature saline for 5 min, while Group B received 20 mL of saline at 2.5°C for 5 min, with the saline stored in a controlled refrigerator and monitored using a digital thermometer. After opening the sealed envelopes, the chosen group irrigation was done, canals were dried with paper points, and calcium hydroxide intracanal medicament was placed. The access cavity was provisionally restored with Cavit, and the patient was recalled for obturation after 7 days. Patients were directed to avoid analgesics unless pain was excruciating, in which case ibuprofen (400 mg every 8–12 h) was recommended. Pain was measured using the VAS at three time points: preoperatively, 24 h postoperatively (postopVAS24) and 48 h post‐treatment (postopVAS48). Data on analgesic consumption were also recorded.

## 3 Statistical Analysis

The statistical analysis was conducted using R (https://www.r‐project.org/) and the Jamovi tool [17]. We performed the Mann‐Whitney *U* Test and the Kruskal‐Wallis Test in order to analyse the pre‐ and post‐operative VAS pain scores between the normal saline and cryotreated saline treatment groups. However, we conducted the normality test (Shapiro‐Wilk) and the homogeneity of variances test (Levene's) to carefully select the type of statistical analysis. Furthermore, the Dwass‐Steel‐Critchlow‐Fligner pairwise comparisons were performed. Data visualisations were generated using R.

## 4 Results

In this study, we used *n* = 88 participants (2 out of the 90 participants were omitted from the study due to failure to continue treatment). The demographic and clinical features included age (years), gender, preop VAS, postop VAS 24, postop VAS 48, analgesics, and group (normal saline and cryotreated saline) for each of the 88 participants. Furthermore, we derived age_group features from the age variable, as well as range_preop VAS, and range_postop VAS features were derived from postop VAS and preop VAS, respectively. The detailed statistical description of all these features used in this study is presented in Table 1. A total of 44 (50%) participants were in the normal saline group and the cryotreated group. The mean age of the participants was 35 ± 9.8 years. A total of 51 (58%) participants were female and 37 (42%) male. All participants reported high preop pain VAS [7‐10], with 59% scoring 9 or 10, indicating severe pain. Postop pain decreased with 78.4% showing 0–3 (low pain) at 24 and 48 h, with 50% reporting no pain (VAS 0) by 48 h. Only 3 (3.4%) participants required an analgesic to control pain.

**TABLE 1:** Statistical description demographic and clinical features used in this study.

Variable name	Categories/values	Summary statistics
Age	Mean ± SD range (min–max)	35.0 ± 9.8 19–59
age_group	18–40	61 (69.3%)
	41–60	27 (30.7%)
gender	F	51 (58.0%)
	M	37 (42.0%)
analgesics	No	85 (96.6%)
	Yes	3 (3.4%)
preopVAS	1:10	**1–6:** 0 (0%); **7:** 18 (20.5%), **8:** 18 (20.5%), **9:** 25 (28.4%), **10:** 27 (30.7%)
postopVAS24	0:10	**0:** 8 (9.1%); **1:** 22 (25.0%); **2:** 24 (27.3%); **3:** 17 (19.3%); **4:** 12 (13.6%); **5:** 3 (3.4%); **6:** 2 (2.3%); **7–10:** 0 (0%)
postopVAS48	0:10	**0:** 44 (50.0%), **1:** 31 (35.2%), **2:** 12 (13.6%), **3:** 1 (1.1%); **4–10:** 0 (0%)
preop_range	7–10	88 (100.0%)
postop_range	0–3	69 (78.4%)
	4–6	18 (20.5%)
	7–10	1 (1.1%)
group	Normal saline	44 (50.0%)
	Cryotreated saline	44 (50.0%)

*Note:* Significance of bold values represent the recorded VAS scores.

The objective of this prospective experimental study was to evaluate if cryotreated saline irrigation reduced post‐operative endodontic pain in patients. Therefore, we conducted a statistical analysis to compare the VAS scores between study groups: Group A (Normal Saline) and Group B (Cryotreated saline). In order to perform the statistical data analysis, we first conducted some assumption checks to identify the statistical test suitable for our data. Therefore, the normality test (Shapiro‐Wilk) and the homogeneity of variances test (Levene's) were performed to check the key assumptions required for using a particular statistical test [18, 19]. The normality test (Shapiro‐Wilk) identifies whether the data in each group are distributed normally (null hypothesis: H_o_). Likewise, the homogeneity of variance test evaluates whether the variances in two groups are equal (null hypothesis: H_o_). The low *p*‐value rejects H0, which suggests the violation of the assumption of normality (in normality test) and equal variance (in homogeneity of variance test). The results in Table 2 indicated that all three variables (preopVAS, postopVAS24 and postopVAS48) significantly violate the normality assumption (*p*‐value < 0.05) and deviate from normality. However, the *p*‐values of the homogeneity of variance test (the *p*‐values > 0.05) indicated no evidence of unequal variances. Consequently, non‐parametric tests such as the Kruskal‐Wallis test and Mann‐Whitney *U* test, which do not assume normality.

**TABLE 2:** Assumption checks for parametric tests: Normality and homogeneity of variance of VAS pain scores across treatment groups.

VAS variable	Normality test (Shapiro–Wilk)		Homogeneity of variances test	
	*p*	Conclusion	*p*	Conclusion
preopVAS	< 0.001	Not normal	0.248	Equal variance
postopVAS24	0.001	Not normal	0.078	Equal variance (marginal)
postopVAS48	< 0.001	Not normal	0.147	Equal variance

*Note:* A low *p*‐value suggests a violation of the assumption of normality (in Normality Test) and a violation of the assumption of equal variances (in Hemogeneity of Variance Test).

The Mann‐Whitney *U* Test and Kruskal‐Wallis Test were performed to compare the VAS scores between study groups (Normal Saline and Cryotreated Saline). The results of both tests are presented in Table 3. The results of the Mann‐Whitney *U* Test revealed that the preop VAS indicated no significant difference between groups (U‐statistic = 854 and *p*‐value = 0.324). However, the postoperative VAS pain scores at 24 h indicated a significant difference between normal saline and cryotreated saline groups with U‐statistic = 665 and *p*‐value = 0.010 (*p*‐value < 0.05). The lower U‐statistic of postopVAS24 as compared to preop VAS (665 vs. 854) suggested a greater difference between the two groups. Furthermore, the postoperative VAS score at 48 h revealed that this difference increased more significantly between groups and the effect size is larger compared to postopVAS24 (U‐statistic = 606 and *p*‐value < 0.001). Overall, the results of the Mann‐Whitney *U* Test reported that there was a significant reduction in VAS pain score with the cryotreated group at both postoperative time points.

**TABLE 3:** Independent Samples *t*‐test comparing pain scores between Group A (Normal saline) and Group B (Cryotreated saline).

VAS variable	Group descriptives	Mann–Whitney *U* Test	Kruskal‐Wallis	U‐Statistic	*p*	*χ* ^2^	*p*
	Group	*N*	Mean/median ± SD				
preopVAS	Normal	44	8.568/9.00 ± 1.169	854	0.324	0.981	0.322
	Cryotreated	44	8.818/9.00 ± 1.063				
postopVAS24	Normal	44	2.659/2.50 ± 1.493	665	**0.010**	6.705	**0.010**
	Cryotreated	44	1.795/2.00 ± 1.19				
postopVAS48	Normal	44	0.932/1.00 ± 0.818	606	**< 0.001**	11.041	**< 0.001**
	Cryotreated	44	0.386/0.00 ± 0.579				

*Note:* Hₐ μ_Normal Saline_ ≠ μ_Cryotreated saline_. Significance of the bold values highlight statistically significant findings.

Abbreviations: SD, standard deviation; VAS, Visual Analogue Scale.

**FIGURE 1:**
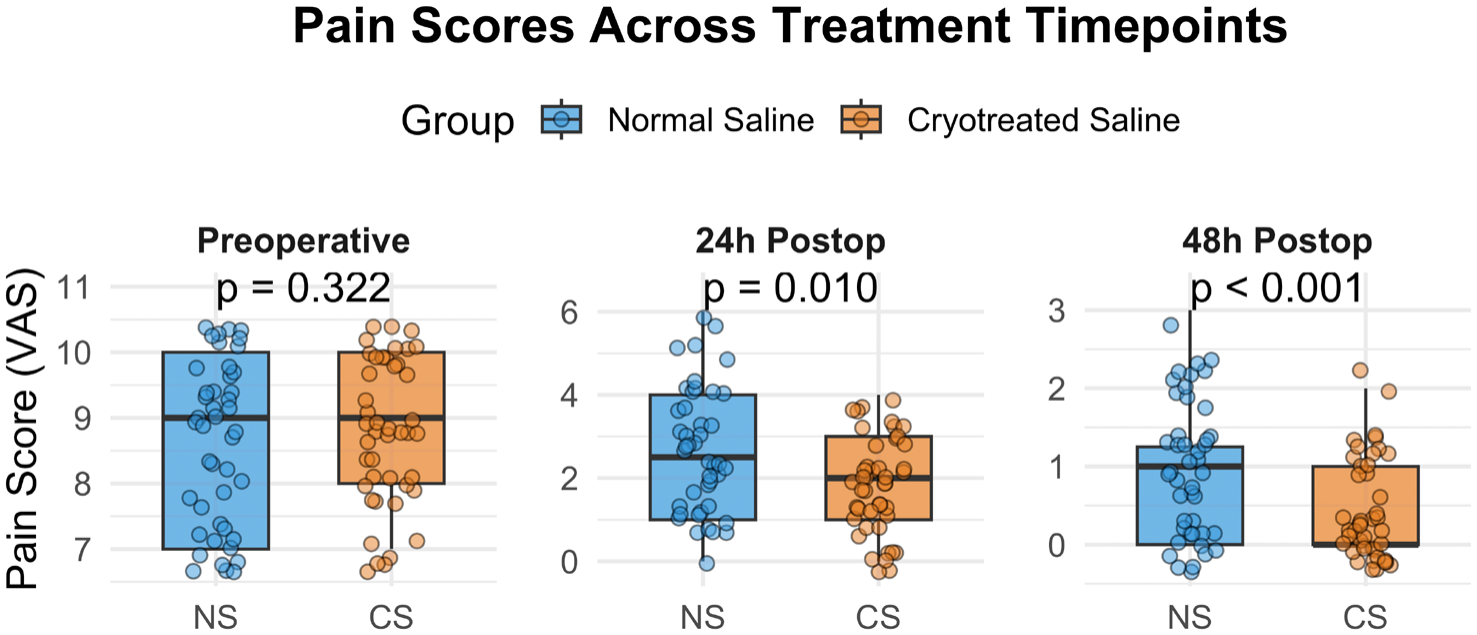
Distribution of VAS pain scores across treatment timepoints with respect to treatment group.

In addition, the results of Kruskal‐Wallis Test (Table 3) indicated that the preop VAS did not differ in the normal and cryotreated groups (*p*‐value = 0.322). The mean and median VAS scores recorded preoperatively were similar across the groups, indicating no significant difference in baseline pain levels. However, the postop VAS 24 reported a significant difference between the normal saline and cryotreated saline groups with a *p*‐value of 0.010 (*p* < 0.05). In addition, the group descriptives such as mean and standard deviation (SD) indicated that the normal saline group reported a greater VAS score as compared to cryotreated saline with 2.659 ± 1.493 versus 1.795 ± 1.193, respectively. Furthermore, the postopVAS48 reported that this difference increased significantly between normal saline and cryotreated saline groups (*p* < 0.001) with mean and SD of 0.932 ± 0.818 vs. 0.386 ± 0.579, respectively. Figure 1 illustrates the distribution of three pain‐related VAS scores with respect to treatment group, including normal saline and cryotreated saline. The boxplot for preoperative VAS pain scores showed that there is no significant difference in VAS pain score between treatment groups. However, the box plots for postoperative VAS pain score at 24 and 48 h illustrated that the cryotreated saline group reported a significantly lower pain compared to the normal saline group.

Furthermore, Table 4 illustrates the results of the Dwass‐Steel‐Critchlow‐Fligner (DSCF) pairwise comparison. The DSCF tests revealed that there was no significant difference in preop VAS pain scores across both groups (normal and cryotreated), and both groups were comparable with a *p*‐value of 0.322. However, the cryotreated saline group indicated a significantly reduced pain score compared to the normal saline group at both postopVAS24 (*p*‐value = 0.010) and postopVAS48 (*p*‐value < 0.001). The negative value of the w‐statistic suggested that the second group (cryotreated group) has higher ranks, and the magnitude of the w‐statistic reflects the size of the effect. This could be seen that the cryotreated group revealed a significantly lower pain score at 24 h (W = −3.66) compared to the normal group. However, the w‐statistics for the cryotreated group at 48 h (W = −4.70) indicated more effect in pain reduction compared to the normal saline group.

**TABLE 4:** Dwass‐Steel‐Critchlow‐Fligner pairwise comparisons.

VAS Variable	Pairwise comparison Group A vs. Group B	W‐Statistic	*p*
preopVAS	Normal Saline vs. Cryotreated Saline	1.40	0.322
postopVAS24	Normal Saline vs. Cryotreated Saline	−3.66	0.010
postopVAS48	Normal Saline vs. Cryotreated Saline	−4.70	< 0.001

In summary, the cryotreated saline group exhibited a more substantial decrease in pain relative to the normal saline group. Furthermore, the results of the study suggested that cryotreated saline was more effective in reducing POP at both 24 and 48 h in symptomatic apical periodontitis patients.

## 5 Discussion

According to the study's findings, the cryotherapy group experienced significantly less discomfort at 24 and 48 h than the normal saline group. In particular, the cryotreated saline group's mean pain reduction at 48 h was 0.386 ± 0.579, considerably less than the normal saline group's 0.932 ± 0.818 (*p*‐value < 0.001). These findings are consistent with other research that shows cryotherapy's potential to lessen POP in SAP treatment, including studies by Gundogdu et al. and Hespanhol et al. [9, 14] Additionally, during the 6‐h follow‐up, Al‐Nahlawi et al. saw a substantial decrease in the cryotherapy group's mean pain scores, compared to significant pain in control and room temperature saline groups [20]. Keskin et al. found cryotherapy decreased interappointment pain in asymptomatic apical periodontitis [21]. However, they contrast Al‐Harthi et al.'s findings, which showed that cryotherapy did not considerably lessen pain in SAP patients [22]. This discrepancy could result from variations in patient demographics, study procedures, and pain assessment schedules as Al‐Harthi et al. included a more inclusive approach, selecting cases of asymptomatic apical periodontitis, which may have diluted the effect of cryotherapy in SAP.

The ideal cryotherapy irrigation duration is yet to be determined. However, the time needed for treatment depends on the depth of the tissue; parts with little muscle and fat should be applied for 3 to 5 min, whereas deeper tissues, such as the hip, need about 20 min [4, 6]. Due to differences in dentine thickness and the quantity and direction of dentinal tubules, cold transmission to the periodontal ligament varies between the apical and coronal regions of the root. Compared to cervical dentine, which has larger and more numerous tubules, apical dentine has more mineralized but fewer tubules, thus permitting more effective cold transmission [6]. Furthermore, compared to multi‐rooted teeth, the apical third of single‐rooted teeth allows for quicker cold transmission.

Remarkably, a comparison study performed in irreversible pulpitis (IP) cases revealed a limited reduction in pain with cryotherapy. Bashir et al. reported that cryotherapy (20 mL saline at 2.5°C) reduced mean VAS pain scores from 8.73 ± 1.2 preoperatively to 0.98 ± 0.9 at 24 h and 0.12 ± 0.4 at 48 h, compared to normal saline (8.47 ± 1.3 preoperatively to 1.51 ± 1.2 at 24 h and 0.27 ± 0.5 at 48 h), with a significant difference at 24 h (*p* = 0.01) but not at 48 h (*p* = 0.104) [16]. Similarly, Alharthi et al. found no significant difference in POP between cryotherapy (10 mL saline at 1.5°C–2.5°C) and room‐temperature saline in single‐canal teeth without periapical pathosis. However, both outperformed a no‐irrigation control at 6, 24 and 48 h (*p* ≤ 0.016) [22]. Studies suggest that cryotherapy may be less effective in IP and more in SAP, most likely due to differences in inflammatory mediators and neural sensitisation. SAP cases are more likely to involve established periapical inflammation, which may respond more robustly to cryotherapy's anti‐inflammatory effects, as observed in studies reporting greater pain reduction in periapical pathosis [14, 21]. Jain et al. evaluated the effect of cryotherapy in mandibular premolars with symptomatic irreversible pulpitis and normal periapical tissues [23]. The results of their study indicated that the incidence and intensity of POP were reduced when cold saline solution is used as the final irrigant in endodontic treatment.

By reducing the local temperature below 14°C, cryotherapy also slows cell metabolism and induces vasoconstriction, which prevents nerve impulses from being transmitted. This decreases the chemical mediators that cause pain to be transmitted [4]. These effects of cold therapy may help explain why pain reduction was only observed after 6–24 h.

The physiological effects of cryotherapy are well‐established and include lowering tissue temperature, slowing nerve conduction velocity and inhibiting the release of pro‐inflammatory mediators such as bradykinin, inflammatory cytokines (IL‐1β) and prostaglandins (PGE_2_) [21, 24]. Additionally, cryotherapy limits blood flow to the area, reducing the accumulation of inflammatory cells and alleviating pain. These mechanisms support its utility as an adjunct to root canal therapy in both SAP and IP cases. The study's findings imply that cryotherapy might work better in situations where periapical inflammation outweighs periapical involvement.

In contrast to the cryotherapy group, the normal saline group showed inadequate reductions in pain levels in our findings. These findings are consistent with the results of Akpinar et al. [25] They achieved the lowest pain scores of 5.26 at 24 h, 4.21 at 48 h, and 3.15 at 72 h post‐op compared to the control group's scores of 17.00, 12.00, and 9.00, respectively, suggesting a trend towards enhanced pain relief with cryotherapy.

The VAS was selected due to its reliability and validity for assessing POP [9]. Prior research has demonstrated that VAS is highly useful in endodontics, as it allows patients to comprehend and quantify pain. A 10 cm line is drawn that represents pain, where zero is no pain and 10 is severe pain [26]. Furthermore, VAS has been widely used in clinical trials involving intracanal cryotherapy, reinforcing its suitability for pain evaluation in this study [10].

In this study, the results demonstrated the reduced dependence on analgesics in the cryotherapy group. Patients in the normal saline group consumed ibuprofen, with 6% requiring the analgesic compared to none in the cryotherapy group. These findings were in accordance with the findings of Gundogdu et al. and Vera et al.; they noted that cryotherapy significantly reduced the need for post‐operative analgesic use in patients with symptomatic apical periodontitis [9, 12].

This study, while yielding encouraging results, is not without its limitations. Although the sample size is adequate for statistical evaluation, future research could benefit from a larger cohort to confirm the findings in various populations. Moreover, the long‐term impacts of cryotherapy on periapical healing have yet to be examined. Additionally, discrepancies in methodologies among studies, including variations in saline temperature and irrigation times, may influence results. Al‐Harthi et al. highlighted the necessity of standardised protocols to enhance reproducibility and reliability in outcomes [22].

## 6 Conclusion

Intracanal cryotherapy has demonstrated a remarkable decrease in POP in patients undergoing endodontic treatment for symptomatic apical periodontitis and contributing to a reduced need for analgesics. Nevertheless, the effectiveness of this treatment may differ based on the specific clinical situation, especially when comparing SAP to irreversible pulpitis. This non‐invasive approach, affordability and low incidence of side effects render cryotherapy a beneficial complement to standard endodontic practices. Future studies should aim to refine cryotherapy techniques and investigate their effects on long‐term treatment results and healing.

## Author Contributions

All authors have contributed significantly to this work, including study design, data collection, analysis, and manuscript preparation. All authors approve the final version of the manuscript and agree to its submission.

## Conflicts of Interest

The authors declare no conflicts of interest.

## Data Availability

The data that support the findings of this study are available from the corresponding author upon reasonable request.
